# Distributive quality assurance and delivery of stereotactic ablative radiotherapy treatments amongst beam matched linear accelerators: A feasibility study

**DOI:** 10.1002/acm2.12567

**Published:** 2019-03-18

**Authors:** James Rijken, Hilmar Schachenmayr, Scott Crowe, Tanya Kairn, Jamie Trapp

**Affiliations:** ^1^ GenesisCare Flinders Private Hospital Bedford Park SA Australia; ^2^ Queensland University of Technology Brisbane QLD Australia; ^3^ GenesisCare Shenton House Joondalup WA Australia; ^4^ Royal Brisbane & Women's Hospital Herston QLD Australia

**Keywords:** beam match, match, SBRT, spine, stereo, VMAT

## Abstract

**Purpose:**

Beam matching occurs on all linacs to some degree and when two are more are matched to each other, patients are able to be transferred between machines. Quality assurance of plans can also be performed “distributively” on any of the matched linacs. The degree to which machines are matched and how this translates to like delivery of plans has been the focus of a number of studies. This concept has not yet been explored for stereotactic techniques which require a higher degree of accuracy. This study proposes beam matching criteria which allows for the distributive delivery and quality assurance of stereotactic body radiotherapy (SBRT) plans.

**Method:**

Two clinically relevant and complex volumetric modulated arc therapy (VMAT) SBRT spine and lung plans were chosen as benchmarking cases. These were delivered on nine previously beam matched linacs with quality assurance performed through ArcCheck and film exposure in the sagittal plane. Measured doses were compared to their treatment planning system predictions through gamma analysis at a range of criteria.

**Results:**

Despite differences in beam match parameters and variations in small fields, all nine linacs produced accurate deliveries with a tight deviation in the population sample. Pass rates were well above suggested tolerances at the recommended gamma criterion. Film was able to detect dose errors to a greater degree than ArcCheck.

**Conclusion:**

Distributive quality assurance and delivery of stereotactic ablative radiotherapy treatments amongst beam matched linacs is certainly feasible provided the linacs are matched to a strict protocol like that suggested in this study and regular quality assurance is performed on the matched fleet. Distributive quality assurance and delivery of SBRT provides the possibility of efficiency gains for physicists as well as treatment staff.

## 1 INTRODUCTION

Quality assurance (QA) of linear accelerators (linacs) aims to ensure that the treatment machine parameters match those acquired during commissioning (which ideally are within manufacturer specifications)^1^, ^2^ and are also matched to the treatment planning system (TPS) model. In this way, the development of machine QA has also been the development of beam matching and thus the definition of both are similar, that is, beam matching of a linac involves tweaking machine and beam characteristics to match reference values, within defined tolerances.^2^, ^3^

When two or more linacs are beam matched to each other it allows for “distributive” QA and patient treatment delivery whereby patients can be transferred between machines and treatment plan QA can be performed on any of the beam matched linacs. This also allows for a considerable reduction in workload related to dosimetric tests in commissioning and can even be performed for linacs with differing heads or multi‐leaf collimators (MLCs).^4^ If the reference beam or treatment planning system (TPS) beam model and the new linac beam are found to be in agreement within a specified tolerance limit for a small subset of profile and percentage depth dose measurements, no further dosimetric measurements for the new machine should be required.^5^ Output factor measurements can then be used as a verification of the beam. Hrbacek et al. were able to quantify factors associated with beam matching linear accelerators and a beam matching tolerance was developed using a one‐dimensional gamma analysis (1%/1 mm) of beam data^5^ (a 1% error in depth–dose curve leads to only a 1.7% of treatment delivery error^6^). Sarkar et al. improved upon this criteria by developing a test package to aid in beam match testing^7^ which involved matching profiles and depth dose curves to within 1%/1 mm as well as their first and second derivatives, as they were found to be “ideally suited to discern any variance between the new beam and existing beam.”

Sjöström et al. considered eight fine beam matched Varian iX linear accelerators and performed beam match analysis by delivering standard plans on all the linacs.^8^ The study showed that matching a new linac to a reference linac does not guarantee that it is matched to the established treatment planning model. Similarly, Gersgjevitsh et al.^9^ demonstrated that a single TPS model could not be supported by their three Elekta linacs which were matched according to the vendor's beam matching criteria, calling for stricter criteria and an additional subset of dosimetric data to be matched to if a single model was to be used. Alternatively, a study by Swamy et al., like Sjöström et al., also considered “fine beam‐matched” Varian linacs and found that from delivery of fifteen volumetric modulated arc therapy (VMAT) plans, the standard deviation in gamma (2%/2 mm) pass rates was 1.00%, demonstrating excellent beam matching in terms of VMAT delivery.^10^ To date there have not been any studies published considering beam matching for stereotactic body radiotherapy (SBRT) and the tighter tolerances and objectives it might require, so the feasibility of distributive QA and treatment delivery of SBRT should thus be assessed and verified.

Stereotactic body radiotherapy describes extracranial treatment techniques which utilize a larger delivery of radiation dose than conventional radiotherapy and in fewer fractions resulting in a higher biological effective dose for the treatment site.^11^ The hypofractionated nature of SBRT treatments provides a unique challenge for beam matched linacs, as any small differences in beam delivery will have a larger effect on the overall treatment. This work investigates the effect of current beam matching procedures on complex SBRT delivery by benchmarking nine nominally matched Elekta linacs against standard plans. This is of particular interest for SBRT spine treatments due to increased beam complexities involved. Of chief consideration is the involvement of small fields which have been shown to exhibit inconsistencies between otherwise dosimetrically matched linacs.^12^

## 2 MATERIALS AND METHOD

The tighter beam matching criteria used by the linacs in this study, which improves upon the vendor beam matching criteria and the criteria set out by Hrbacek et al.,^5^ is listed in Table 1. In addition, beam matching had been verified through level 3 testing,^13^ level 3 audits in a variety of heterogeneous phantom with plans ranging from conformal radiotherapy to VMAT SBRT, standard IMRT cases^14^ as well as an ensemble of VMAT benchmarking cases representing ordinary fractionated radiotherapy treatments such as prostate, breast, lung, and head & neck sites.

**Table 1:** Beam matching criteria currently used for Elekta linacs with Agility heads in this study. Each parameter is compared to a reference. Profile points are taken to be within 80% of the field size

Modality	Parameter	Tolerance
Photons	PDD_20,10_	0.5%
	PDD points	0.5%
	Profile points	1.0%
	Wedge profile points	2.0%
Electrons	R50	0.5 mm
	Profile points	1.0%

Two clinical SBRT plans, one spine and one lung, were chosen as benchmarking cases for assessment of SBRT beam matching. Both were previously planned in Pinnacle^3®^ 9.10 (Koninklijke Philips N.V., Amsterdam, The Netherlands) and treated on an Elekta VersaHD^®^ linear accelerator (Elekta, Stockholm, Sweden). The cases were chosen as they were particularly complex compared to other SBRT plans of the same site according to their QA pass rates.^15^ These plans were sent to the respective record and verify systems for treatment.

**Figure 1:**
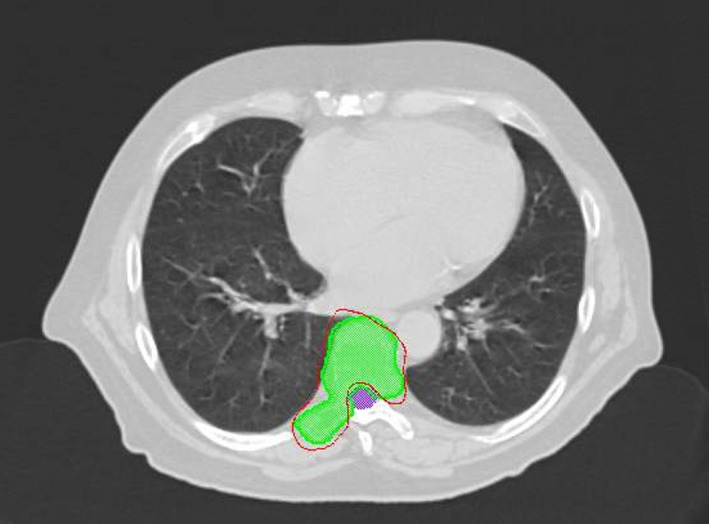
Transverse slice of the original stereotactic body radiotherapy spine plan with the 95% isodose shown in red and the target and spinal cord in green and purple respectively.

**Figure 2:**
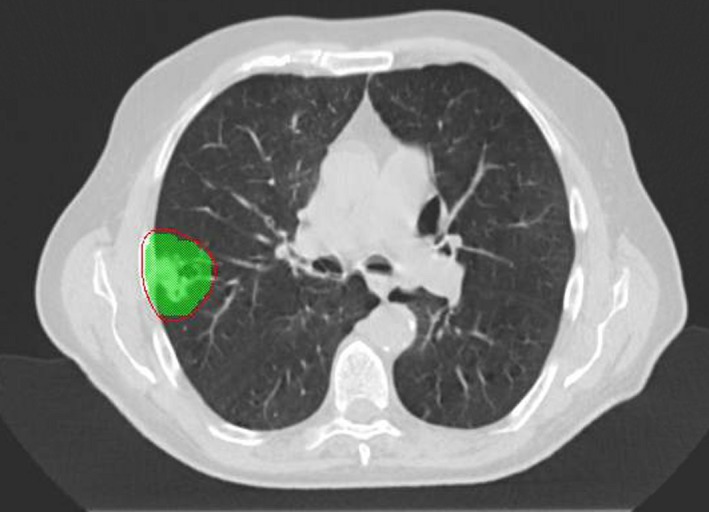
Transverse slice of the original stereotactic body radiotherapy lung plan with the 95% isodose shown in red and the target green.

The spine case included four VMAT arcs with 120 control points per arc and a prescription of 30 Gy in three fractions to the planned target volume and a maximum dose to the spinal cord of 18 Gy.^16^ A transverse slice of the original plan is shown in Fig. 1. The lung case contained 2 VMAT arcs with 90 control points per arc with a prescription of 54 Gy in three fractions and a dose constraint to the lung of V20Gy < 10%. The original plan is similarly shown in Fig. 2. Both cases were planned with the 6 MV modality and calculated on a 2 mm dose grid (1 mm interpolation in SNC Patient software).

A total of nine treatment machines, a mixture of VersaHD^®^ and Synergy^®^ linacs all with Agility^®^ heads, participated in the SBRT benchmark testing. QA was performed on the spine and lung cases with both the ArcCheck^®^ device (Sun Nuclear Corp., Melbourne, FL, USA) and with film in the sagittal plane (using the ArcCheck as a phantom). Table 2 lists the nine linacs as well as their nominally matched beam parameters.

**Table 2:** Matched beam parameters for linacs 1 to 9. Profiles assessed for a 30 × 30 cm^2^ field at 10 cm depth in the in‐plane (IP) and cross‐plane (XP) directions. PDDs are taken for a 10 × 10 cm^2^ field at either 100 or 90 cm SSD for beam quality matching

Linac		Flatness (%)		Symmetry (%)		Quality (90 SSD)		Quality (100 SSD)		Output
#	Model	IP	XP	IP	XP	PDD_20,10_	TPR_20,10_	PDD_20,10_	TPR_20,10_	(cGy/MU)
1	VersaHD	103.8	104.4	100.3	100.8	0.574	0.666			0.996
2	Synergy	103.9	104.0	100.1	100.1			0.588	0.684	1.003
3	VersaHD	104.8	104.4	101.1	100.6	0.574	0.669			0.998
4	VersaHD	103.8	103.7	100.0	100.0	0.573	0.665			0.996
5	Synergy	104.3	104.5	100.7	100.8			0.585	0.682	1.005
6	VersaHD	105.1	104.9	100.9	100.7	0.576	0.669	0.589	0.687	0.995
7	Synergy	104.0	103.8	100.8	100.7			0.588	0.685	1.000
8	Synergy	104.1	103.9	100.7	100.1			0.587	0.684	1.005
9	VersaHD	104.0	103.9	100.3	100.4			0.572	0.665	1.000
*Standard deviation*		*0.5*	*0.4*	*0.4*	*0.3*	*0.002*	*0.003*	*0.007*	*0.009*	*0.004*
*Baseline*		*104.8*	*104.4*	*100.0*	*100.0*	*0.574*	*0.669*	*0.588*	*0.685*	*1.000*

TPR: tissue phantom ratio.

The film utilized was Garchromic™ EBT3 (Ashland Specialty Ingredients, Bridgewater, NJ, USA) and was scanned on either an Expression™ 10000XL or Perfection TM V850 Pro scanner (Epson^®^, Nagano, Japan). 10 Gy reference films were also acquired before quality assurance in order to scale the film dose slightly as required (daily output was also considered). Film .tiff files were processed in ImageJ (NIH, USA) using a rational dose calibration curve according to the methods of Micke et al.^17^ Analysis for both ArcCheck and film data was performed through SNC Patient Software 6.1 (Sun Nuclear Corp., Melbourne, FL, USA) by comparison to TPS exports of RTPLAN and RTDOSE dicom data and two‐dimensional dose planes respectively. All gamma analyses in this study were performed through absolute dose comparison with a global *γ* calculation and a 10% low dose cut‐off threshold.^18^ Global normalization was used as it has been deemed more relevant than local normalization for QA of clinical cases.^19^

In order to observe trends between nominally beam matched machines and assess suitability of moving SABR plans or QA between linacs, multiple distance‐to‐agreement (DTA) criteria were assessed and compared. Other characteristics were also quantified such as the dose gradient between target and central nervous system (CNS) and the CNS point dose difference in the case of spine as well as some machine settings like MLC offsets. Differences were quantified through examination of the standard deviations in the linac population sample.

## 3 RESULTS

**Figure 3:**
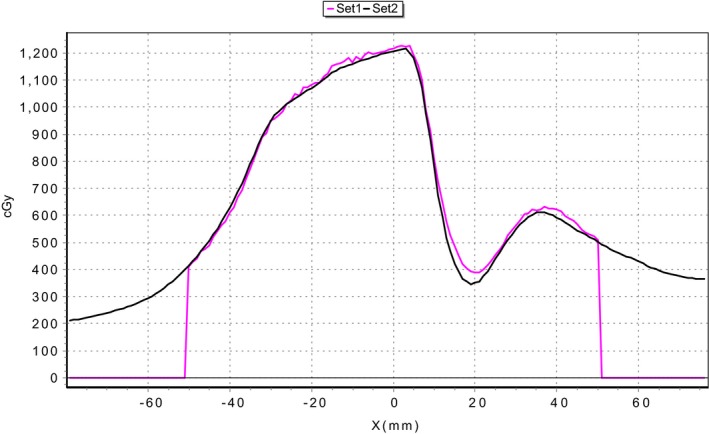
An example of comparison between measured (Set 1) and planned (set 2) sagittal plane dose profiles for the spine case delivered on linac 7. The two‐dimensional dose map comparison had a pass rate of 98.8% at 3%/2 mm

Both spine and lung cases passed ArcCheck and film QA on all machines tested according to routine clinical methods and recommended tolerances (>90% pass rate at 3%/2 mm).^19^ The suggested gamma criteria of 3%/2 mm was used as the reference criterion for comparison between linacs for both film and ArcCheck. An example of a film dose comparison for the spine case assessed at 3%/2 mm is shown in Fig. 3 where the difference in dose gradient between delivered and planned (5.42%/mm) dose can be observed.

QA results are listed in Table 3 for mean gamma pass rates, CNS dose differences and dose gradients. The CNS dose difference was taken as the point dose difference between the TPS and measured doses at the centre of the CNS contour. The CNS dose is important in terms of myelopathy risk^20^ while varying degrees of PTV coverage may be more acceptable to the clinician in terms of accepted protocols and reference trials.^15^, ^21^, ^22^ The dose gradient has been recorded in % dose per millimetre in accordance with similar studies.^23^ The mean values for all four metrics were certainly adequate and standard deviations small, demonstrating excellent beam matching in terms of SBRT delivery.

**Table 3:** Quality assurance results of both spine and lung cases

Metric	Spine case		Lung case	
	Mean	1 SD	Mean	1 SD
ArcCheck gamma pass rate (3%/2 mm)	98.24%	1.39%	98.63%	0.98%
Film gamma pass rate (3%/2 mm)	97.36%	1.53%	96.68%	2.56%
CNS dose difference	2.51%	1.62%	NA	NA
Dose gradient PTV‐CNS (%/mm)	5.49	0.15	NA	NA

CNS: central nervous system.

**Figure 4:**
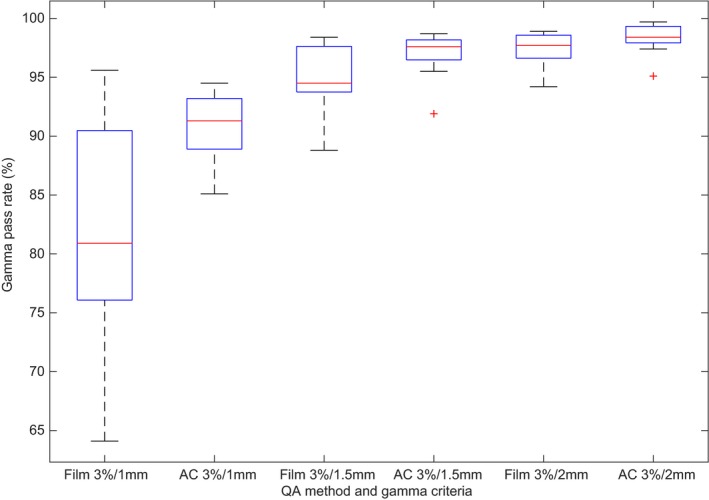
Distribution of film and ArcCheck (AC) quality assurance pass rates per gamma criterion for the spine case (n = 9)

**Figure 5:**
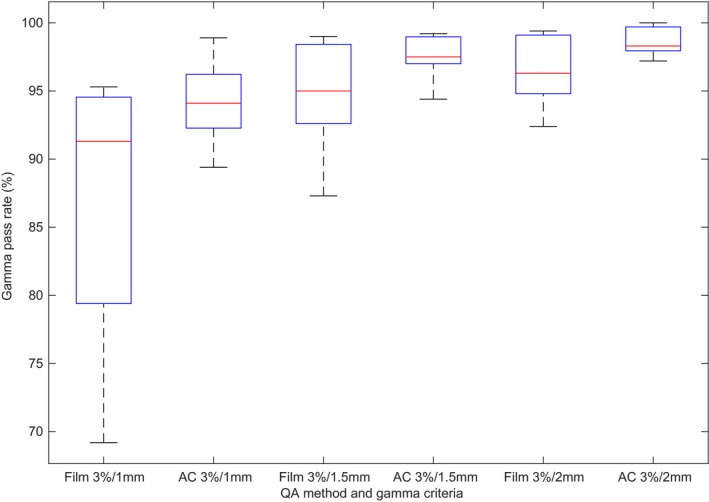
Distribution of film and ArcCheck (AC) quality assurance pass rates per gamma criterion for the lung case (n = 9)

More sensitive DTA of 3%/1.5 mm and 3%/1 mm were used in Figs. 4 and 5, demonstrating the distribution of QA results from the spine and lung cases for both film and ArcCheck. Minimum and maximum results are represented by the whiskers with outliers depicted as crosses. The box shows the interquartile with the line therein representing the median value. As the DTA criteria is tightened, not only do the median pass rates decrease but the distribution of results widens. Of particular note is the difference in distribution and thus sensitivity between the film and ArcCheck QA methods, with ArcCheck demonstrating a poorer ability to detect dose errors compared to film.

## 4 DISCUSSION

In the literature, the quality or accuracy of beam matching has been assessed by the standard deviation in gamma pass rates for a particular treatment,^10^ with standard deviations around 1% constituting excellent agreement. Accordingly, this study has shown that the linacs considered are also well matched in parameters (output factors, depth‐dose curves, profiles, energy and dose rate, MLC positional accuracy, MLC speed, jaw positional accuracy, gantry speed etc.^3^) by demonstrating standard deviations of 1.53% & 2.56% (film) and 1.39% & 0.98% (ArcCheck) for the complex SBRT spine and lung cases, respectively (gamma criterion of 3%/2 mm). In addition, the passing result on all linacs for the two example cases provides a positive appraisal of the state of beam matching among the Elekta linacs in this study. This result also provides some evidence that the inconsistencies in small field sizes, in otherwise beam‐matched linacs, do not produce appreciable differences in treatment delivery accuracy for treatments such as SBRT that utilize a high proportion of small fields.^12^

CNS doses for the spine case also show good agreement amongst the linac sample with a difference to planned dose of 2.51% ± 1.62%. In an absolute sense, this also satisfies the recommendations of Palta et al. where the action limit for low dose, low gradient regions is up to 7%.^24^ All target doses were within 5% of planned.^25^, ^26^, ^27^ Aside from the CNS dose, the dose drop off between target and spinal cord is one of the most important aspects of SBRT spine QA. The variation in dose gradient was also found to be very low with a standard deviation of 0.15%/mm which puts the mean measured result within uncertainty of the planned 5.42%/mm. A dose gradient of 5.42% is within the bounds of gradients observed in other SBRT spine studies.^23^

Considering just the mean gamma pass rate and its standard deviation as its uncertainty, then the accuracy of a distributive quality assurance result (at 3%/2 mm) can be assured to be an accurate representation of the treatment delivery accuracy to within 1.5% for spine and 2.6% for lung cases when QA is performed with film. ArcCheck, while not used routinely used for SBRT spine and lung QA, demonstrates similar results to film with the uncertainty in pass rate well within AAPM TG‐218 tolerances of 90% at 3%/2 mm^19^ (Table 3). In order to detect subtler erroneous segment delivery or to discern the degree of systematic or random errors, an analysis with tighter gamma criteria is recommended.^19^ Larger variations at the stricter DTA criteria of 1 mm observed in all methods of QA (Figs. 4 and 5) demonstrate there are differences yet between nominally beam matched linacs, though differences that are not enough to produce significant disagreements in quality assurance results at recommended gamma criteria. Rather, they are partly indicative of the random and thus unmatchable nature of MLC position discrepancies, the effects of which are expected to be exhibited for complex treatment plans such as those tested in this study.^15^, ^28^

While both methods of QA attempt to capture the accuracy of treatment delivery, ArcCheck and film do not measure the exact same parameters. Film is concerned with a single in‐phantom dose plane while ArcCheck attempts to gain an appreciation of the total dose fluence. The two QA methods also differ in accuracy on a few levels. Film receives a higher maximum dose so is less sensitive to low dose differences (when using global comparison), suffers from processing and calibration uncertainties but has a higher resolution (scanned at 75 DPI) and can show where dose differences will likely occur anatomically. ArcCheck receives a lower maximum dose due to the fluence washout so is arguably more sensitive in regions with low dose fluence, can detect angular dependent fluence errors, has lower uncertainty in dose calibration but also has a lower resolution (detector spacing of 1 cm^29^). Some difference in pass rates can thus be expected.

On close inspection of Figs. 4 and 5, there is indeed a discrepancy in the range of film and ArcCheck results for a given DTA, with a much larger spread in the film results. So, while it is true that the trends in ArcCheck and film results are well‐correlated for SBRT,^15^ at the more sensitive DTA of 1 mm it is clear that ArcCheck's lower resolution masks some of the random errors that are detectable by film. ArcCheck may therefore provide misleading results in the case of SBRT if one is concerned with tighter DTA. Certainly, it is not enough to just have a gamma criterion of 3%/2 mm^19^ for the ArcCheck device in the case of VMAT SBRT without the addition of film to support a pass rate >90% when performing SBRT benchmarking by verifying the in‐phantom target and CNS doses.

Considering individual machines, linac 4 and 9 were initially outliers in results, failing in the case of spine. Despite performing ordinary VMAT QA to a satisfactory level of accuracy, dose profiles for the two SBRT cases initially appeared either shrunk or stretched. In these cases, the jaws and MLCs were recalibrated. The recalibration process, called the “Agility Workflow,” was avoided in linacs 1 and 5 by instead applying sub‐millimetre MLC offsets to their MLC calibrations. Both methods improved SBRT performance to produce passing results. These results suggest that additional criteria are required for SBRT beam matching — simply to perform complex benchmarking cases such as those demonstrated in this study to observe any need for MLC/recalibration or offsets. This also indicates that, if a distributive QA and treatment delivery model is adopted, a weekly or monthly complex SBRT plan QA is required on all linacs to ensure that MLC calibration has not drifted. If the SBRT workload is high enough then each machine may perform enough QA weekly to avoid the need for this, but if the SBRT patient workload is light then this may stretch physics resources ‐ especially if variations in bunker designs require a pretreatment run through of beams (e.g., to avoid couch collision with couch rotations).

Interestingly, Table 2 shows that the profile flatnesses are not tightly grouped about the baseline value, making use of their 1% wiggle room. This appears to have little effect on the QA results, perhaps for the sole reason that the test cases involved small, isocentric fields where profiles become increasingly indistinguishable, even between flattened and unflattened fields. Even for nonisocentric plans, the 3% dose difference criterion would not be sensitive enough to detect these profile differences. This then puts greater emphasis in the accuracy of the machine's MLC calibration and dose output.

In addition to the tests described here, other parameters need to be tighter than for regular treatments^30^ such as the geometric accuracy of linac and couch as well as image guidance systems. These should also be re‐evaluated before each treatment. The tests in this study were concerned with doses calculated in homogeneous water equivalent phantoms, so the dose calculation accuracy in heterogeneous anatomical phantoms should also be verified in a method such as that described in the beam matching process for the Elekta linacs in this study.

This study has shown that, for nine nominally matched Elekta linacs, SBRT spine and lung delivery was equivalent among linacs and well within accepted tolerances. This sample of linacs is thus a good candidate for moving toward the feasible goal of a distributive QA and treatment. The clinical impact of the calculated uncertainties is certainly within accepted limits.^26^, ^27^

For a department that wishes to beam match their linear accelerator fleet to a degree beyond VMAT that allows for distributive QA and delivery of SBRT, the following items are recommended:

1. Profiles and PDDs should be matched to criteria that is stricter than vendor guidelines.^9^ For example, the linacs in this study were matched at commissioning with PDD_20,10_ matching <0.5% and all points in the PDD within 0.5% of the reference curve. All open profile points within 80% of the field size were within 1% of reference
2. Comprehensive VMAT benchmarking should be performed for a variety of treatment sites, verifying a base level of beam matching traceable to the TPS model.^7^ Level 3 testing should also be performed in anatomical phantoms.
3. kV‐MV isocentre coincidence should be within specification for SBRT delivery for all linacs concerned. Ideally, this should be checked regularly. Image guidance, immobilization, and linac capabilities should, of course, be equivalent between machines.
4. Complex SBRT plans can be used as benchmark cases with QA performed with film (and diode array) at a range of gamma criteria. If cases fail, then the jaw or MLCs may need to be recalibrated of offsets applied to the existing calibration. A diode array in not sufficient in itself for accurate benchmarking
5. QA results should all pass film QA with a small standard deviation, thus demonstrating a low uncertainty in accuracy for distributive QA and treatment delivery.
6. If the SBRT workload is light, then regular testing of each machine through a standard complex SBRT case should be performed to observe any drift in MLC calibration.
7. Daily output and imaging checks should be performed during run‐up or otherwise to capture any gross differences on the day of treatment.

## 5 CONCLUSION

A study of complex SBRT lung and spine cases was conducted through delivery and quality assurance on nine beam matched linacs in order to assess the feasibility of a distributive approach to SBRT QA and delivery. The results of this study suggest that at recommended gamma criterion the delivery accuracy of the SBRT cases is well‐matched between machines and well above the recommended gamma pass rates, with differences in pass rates at stricter criteria attributed to MLC discrepancies. While both film and ArcCheck are informative in the benchmarking process, film should always be used for QA of clinical plans. Distributive QA and delivery of SBRT is thus feasible but requires initial beam matching of machines at stricter criteria than recommended by manufacturers as well as an ongoing QA program that also includes assessments of other parameters like image guidance.

## CONFLICTS OF INTEREST

None.
